# hSulf-1 inhibits cell proliferation and migration and promotes apoptosis by suppressing stat3 signaling in hepatocellular carcinoma

**DOI:** 10.3892/ol.2014.1848

**Published:** 2014-02-03

**Authors:** LING LIU, FENG DING, JIWEI CHEN, BOYONG WANG, ZHISU LIU

**Affiliations:** 1 Department of General Surgery, Zhongnan Hospital of Wuhan University, Wuhan, Hubei 430071, P.R. China; 2 Department of Clinical Laboratory, Wuhan Puai Hospital, Wuhan, Hubei 430033, P.R. China

**Keywords:** human sulfatase-1, hepatocellular carcinoma, stat3 signaling

## Abstract

Human sulfatase-1 (hSulf-1) has been shown to desulfate cellular heparin sulfate proteoglycans and modulate several growth factors and cytokines. However, hSulf-1 has not been previously shown to mediate the signal transducer and activator of transcription 3 (stat3) signaling pathway, which is known to regulate cell proliferation, motility and apoptosis. The present study investigated the role of hSulf-1 in stat3 signaling in hepatocellular cancer. hSulf-1 expression vector and stat3 small interfering RNA (siRNA) were constructed to control the expression of hSulf-1 and stat3 in HepG2 cells. hSulf-1 was found to inhibit the phosphorylation of stat3 and downregulate its targeted protein. MTT and Transwell chamber assays, as well as Annexin V/propidium iodide double-staining methods, were used to examine the effects of hSulf-1 on stat3-mediated motility, proliferation and apoptosis in HepG2 cells. Transfection with hSulf-1 cDNA and/or stat3 siRNA inhibited cell proliferation and motility, concurrent with G_0_/G_1_ and G_2_/M phase cell cycle arrest and apoptosis. Overall, the results of the current study suggested that hSulf-1 functions as a negative regulator of proliferation and migration and as a positive regulator of apoptosis in hepatocellular carcinoma, at least partly via the downregulation of stat3 signaling.

## Introduction

Hepatocellular carcinoma (HCC) is one of most common causes of cancer-related mortality worldwide and the incidence of this cancer has been increasing in recent years (1,2). Improvements in survival are likely to depend on an improved understanding of the molecular processes involved in tumorigenesis and metastasis in hepatocellular cancer.

Human sulfatase 1 (hSulf-1), previously characterized as a heparin-degrading endosulfatase, negatively regulates growth factor and cytokine signaling and proteolysis by desulfation of cell surface heparin sulfate proteoglycans (HSPGs), major constituents of the extracellular matrix (3–6). This biological effect requires sulfation of defined sites on glycosaminoglycan chains. Previous studies have demonstrated that hSulf-1 is downregulated in cancer cell lines originating from various types of human cancer, including ovarian, breast, renal and HCC, and that its expression is regulated by epigenetic mechanisms, including DNA methylation and histone acetylation (7–10).

Additionally, previous studies have demonstrated that re-expression of hSulf-1 in ovarian cells suppresses fibroblast growth factor (FGF)-2 and heparin-binding epidermal growth factor (HB-EGF) signaling and inhibits cell proliferation and invasion *in vitro (*11–14). Further studies of the role of hSulf-1 in tumorigenesis and progression have also found that expression of hSulf-1 inhibits hepatocyte growth factor (HGF) and vascular endothelial growth factor (VEGF) signaling (15–18). While signal transducer and activator of transcription 3 (stat3) signaling is known to be activated by several growth factor receptors, including epidermal growth factor receptor (EGFR) and platelet-derived growth factor receptor (PDGFR), which are important in cell proliferation, migration, apoptosis and angiogenesis (19–20), no previous studies have found a role for hSulf-1 in regulating the stat3 signaling pathway.

Since the stat3 signaling pathway is one of the most important signal transduction cascades characterized to date and is known to be involved in the regulation of cytokine receptor signaling in HCC (21–22), the potential link between hSulf-1 and stat3 in HCC must be investigated. Thus, in the current study, to confirm the role of hSulf-1 in the proliferation, migration and apoptosis of HCC, hSulf-1 was re-expressed in HCC cells and stat3 siRNA was constructed to manipulate the expression of this critical signaling molecule *in vitro*. In addition, the present study sought to determine whether the stat3 pathway is mediated by HGF, a potent mitogen and key regulator of cell proliferation, differentiation and motility in HCC (23–25), in HCC cells exhibiting differential expression of hSulf-1. The results indicated that hSulf-1 may inhibit HCC growth and migration through suppression of the stat3 signaling pathway and that the antiproliferative activity of hSulf-1 in HepG2 cells is due to cell cycle arrest and apoptosis.

## Materials and methods

### Plasmids and siRNA

The plasmid containing whole-length hSulf-1 complementary DNA was purchased from Wuhan Genesil Biotechnology Co., Ltd. (Wuhan, China) and stat3 siRNA was purchased from Origene (Rockville, MD, USA). The stat3 siRNA target sequence used was GGCGTCCAGTTC ACTACTA and the control siRNA target sequence used was AATTCTCCGAACGTGTCACGT. Transfections were performed using Lipofectamine 2000 (Invitrogen Life Technologies, Carlsbad, CA, USA).

### Cell culture

The HCC cell lines, HepG2, Hep3B, Huh-7 and SMMC-7221, were purchased from Shanghai Cell Bank (Shanghai, China) and were cultured in media according to the manufacturer’s instructions. HepG2 cells stably expressing hSulf-1 were selected with 600 μg/ml G418 (Invitrogen Life Technologies) and the transfection results were detected by western blotting. Cells were maintained at 37°C in an atmosphere of humidified air with 5% CO_2_.

### Treatment of cells with trichostatin A (TSA) and 5-aza-dC

All drugs were added the day after the subculture of HepG2 cells. The 5-aza-dC (Sigma-Aldrich, St. Louis, MO, USA) was added at concentrations of 0, 0.5, 1.0, 2.5 and 5.0 μmol/l at 24, 48 and 72 h time points. For TSA (Sigma-Aldrich) treatment, HepG2 cells were treated with TSA concentrations of 0, 0.25 and 0.5 μmol/l and cells were incubated for 24 h. For combined treatment, HepG2 cells were treated with 5.0 μmol/l 5-aza-dC at 24 and 48 h time points and then with 0.5 μmol/l TSA for 24 h. When HepG2 cells were treated with no drug, identical volumes of water were added.

### RNA extraction and semi-quantitative reverse transcription-polymerase chain reaction (RT-PCR)

Total RNA was isolated from HCC cells using an RNeasy kit (Qiagen, Valencia, CA, USA). Taq enzyme and PCR reagents were purchased from Tiangen Corporation (Beijing, China). Primers for amplifying *hSulf-1* and *GAPDH*, which was used as internal control in RT-PCR, were purchased from Sangon Corporation (Shanghai, China). The forward and reverse primers used were as follows: 5′-CTCACAGTCCGGAGCGGAAC-3′ (forward) and 5′-CACGGCGTTGCTGCTATCTGCCAGCATCC-3′ (reverse) for *hSulf-1*; and 5′-AGTCAACGGATTTGGTCGT-3′ (forward) and 5′-TTGATTTTGGAGGGATCTG-3′ (reverse) for *GAPDH.* The primers yielded amplicons of 371 and 238 bp, respectively. The PCR conditions used were as follows: 94°C for 5 min, followed by 34 cycles of 15 sec at 94°C, 30 sec at 62°C and 30 sec at 72°C, followed by a final extension at 72°C for 10 min. Semi-quantitative RT-PCR products were analyzed on 1% agarose gels stained with ethidium bromide.

### Western blotting

HepG2 cells were lysed in RIPA buffer (Beyotime Institute of Biotechnology, Shanghai, China). Cell lysates (20 μg protein/lane) were loaded and separated on gradient polyacrylamide gels and then transferred to polyvinylidene difluoride membranes by electroblotting (Millipore Corp., Boston, MA, USA). Following blocking with 5% non-fat milk containing 0.3% Tween 20 for 1 h, the membranes were incubated overnight with primary antibodies at 4°C, including anti-hSulf-1 (1:250), -stat3 (1:500), -phospho-stat3 (1:500), -phospho-c-met (1:500), -bcl-2 (1:1000) and -cyclin D1 (1:500) (Santa Cruz Biotechnology, Inc., Santa Cruz, CA, USA). The membranes were washed three times with Tris-buffered saline containing Tween 20 and membranes were then incubated with horseradish peroxidase-conjugated secondary antibodies (R&D Systems China Co., Ltd., Shanghai, China) at 4°C for 1 h. Subsequently, membranes were exposed to enhanced chemiluminescent reagents for detection of protein bands. β-actin was used as an internal control.

### Cell proliferation analysis

Cell proliferation was measured using an MTT assay (Sigma-Aldrich). Cells were harvested and plated in 96-well plates at 4×10^3^ cells/well in 100 ml culture medium and then maintained at 37°C in an incubator containing 5% CO_2_ for three days. In total, 20 μl MTT dye was added to each well (5 mg/ml). After 4 h of incubation, 100 μl dimethyl sulfoxide was added for 10 min to dissolve the crystals. The absorbance was measured by a microtiter plate reader at 490 nm (no. DG5033A, Jinggong Industrial Co., Ltd., Shanghai, China). Cell viability was expressed as an optical density value.

### Transwell chamber assay

Migration was detected by the Transwell chamber assay. A total of 5×10^5^ cells per ml were starved overnight in serum-free medium. In total, 100 μl of cells were then added to each upper well in a 24-well Transwell plate (8.0-μm pore size; Corning, Inc., Cambridge, MA, USA) and medium containing 10% fetal bovine serum (600 μl) was added to the lower well. Cells were incubated in the Transwell chambers for 24 h. Then, the Transwells were extracted, the medium in the upper well was removed and the Transwells were washed in phosphate-buffered saline (PBS) once. The residual cells in the upper well were swabbed and stained with 0.5% crystal violet for 20 min. Cells that had migrated through the Transwell were dissolved in 10% acetic acid and the absorbance was measured at 560 nm.

### Cell cycle analysis

Cells were seeded at a density of ~6×10^5^ cells/ml and treated with 5 μmol/l cisplatin to determine the effects of hSulf-1 on cisplatin-induced cell cycle arrest for 24 h. Following incubation, cells were washed with PBS and fixed with 70% ethanol overnight at 4°C. Next, cells were stained with 1 ml propidium iodide (PI, Sigma-Aldrich) synthetic dye solution (20 μg/ml PI, 20 μg/ml RNase, 0.5% Triton X-100 and 1 g/ml sodium citrate) for 30 min at 37°C in the dark and then analyzed by flow cytometry using an FC 500 MPL instrument (Beckman Coulter, Miami, FL, USA). The cell number in each phase in every group was calculated using ModFit software (Verity Software House Corp., Topsham, ME, USA).

### Cellular apoptosis assay

Cells were plated at a density of 6×10^5^ cells/ml. Following treatment with 5 μmol/l cisplatin, apoptotic cells were quantified by Annexin V/PI double staining (Jingmei Biotech Co., Ltd., Shenzhen, China). The double-staining technique was performed as follows, according to the manufacturer’s instructions. Cisplatin-treated cells were collected and then washed twice in cold PBS. Cell pellets were resuspended in 250 μl 1X binding buffer (Jingmei Biotech Co., Ltd.) and resuspended cells were gently vortexed and stained with 5 μl Annexin V-fluorescein isothiocyanate and 10 μl PI for 15 min in the dark at room temperature. The results were analyzed using flow cytometry (PC 500 MPL, Beckmann Coulter, Miami, FL, USA) according to the manufacturer’s instructions.

### Statistical analysis

All data obtained in triplicate independent experiments were evaluated using GraphPad Prism 5.02 for Windows (GraphPad Software, Inc., La Jolla, CA, USA). Data are expressed as the mean ± standard error. The significance of differences between groups was determined by two-sided t-tests. P<0.05 was considered to indicate a statistically significant difference.

## Results

### Expression of hSulf-1 mRNA decreases in HCC cell lines and reactivates with 5-aza-dC and/or TSA in HepG2 cells

**Figure 1:**
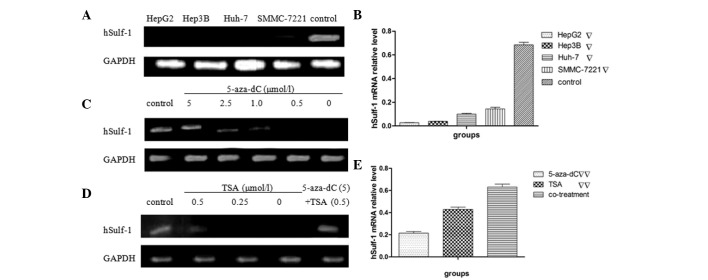
Expression of hSulf-1 in HCC cell lines following treatment with 5-aza-dC and/or TSA. (A and B) Semi-quantitative reverse transcription-polymerase chain reaction analysis of four human HCC cell lines (HepG2, Hep3B, Huh-7 and SMMC-7221) indicated that all HCC cell lines tested express low levels of hSulf-1 mRNA. (C, D and E) Expression of hSulf-1 increased when treated with 5-aza-dC or TSA in HepG2 cells and also increased due to a synergistic effect of 5.0 μmol/l 5-aza-dC and 0.5 μmol/l TSA combined treatment. ^▽^P<0.05, vs. control group and ^▽▽^P<0.05, vs. co-treatment group. Control group, parental cells; hSulf-1, human sulfatase-1; HCC, hepatocellular carcinoma; TSA, trichostatin A.

Firstly, the expression of *hSulf-1* mRNA was evaluated in four human HCC cell lines (HepG2, Hep3B, Huh-7 and SMMC-7221) by semi-quantitative RT-PCR. All the HCC cell lines tested were found to express low or undetectable levels of hSulf-1 mRNA (Fig. 1A and B). The expression of hSulf-1 increased when treated with 5-aza-dC or TSA in HepG2 cells. In addition, hSulf-1 may be reactivated significantly with the appropriate concentration of 5.0 μmol/l 5-aza-dC and 0.5 μmol/l TSA. The expression of hSulf-1 also increased due to a synergistic effect of 5.0 μmol/l 5-aza-dC and 0.5 μmol/l TSA combined treatment (Fig. 1C–E).

### hSulf-1 inhibits the phosphorylation of stat3 in HepG2 cells

**Figure 2:**
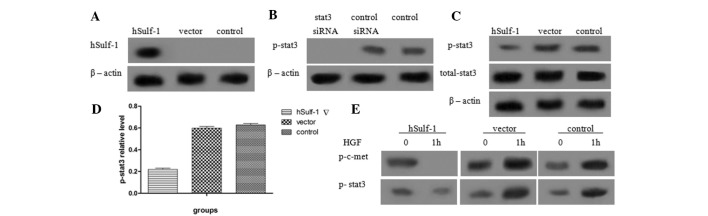
Effects of hSulf-1 on stat3 signaling in HepG2 cells. (A and B) Expression of the indicated factors was examined by western blotting. (C and D) The phosphorylation of stat3 and expression of total stat3 protein were analyzed by western blotting followed by densitometry in hSulf-1 and empty vector transfected cells. The values indicated the ratio of phospho-stat3/β-actin. β-actin was used as the loading control. (E) Following treatment with 5 ng/ml hepatocyte growth factor, the phosphorylation of c-met and stat3 increased. However, this effect was reversed in hSulf-1-transfected HepG2 cells. ^▽^P<0.05, vs. control group or vector group. Control group, parental cells; vector group, empty vector-transfected cells; hSulf-1 group, hSulf-1-transfected cells. hSulf-1, human sulfatase-1; stat3, signal transducer and activator of transcription 3.

Previous studies have shown that dysregulation of the stat3 signaling pathway is involved with HCC development and metastasis. Therefore, the effects of hSulf-1 on the stat3 signaling pathway were investigated in HepG2 cells. The expression of hSulf-1 was detected in hSulf-1-transfected HepG2 cells and HepG2 cells transfected with empty vector. Additionally, the phosphorylation of stat3 in hSulf-1-transfected HepG2 cells and control siRNA-transfected HepG2 cells was examined (Fig. 2A and B). The results showed that overexpression of hSulf-1 reduced the phosphorylation of stat3 in HepG2 cells, but did not affect the expression of total stat3 (Fig. 2C and D). Furthermore, when hSulf-1-negative HepG2 cells were treated with 5 ng/ml HGF, the phosphorylation of c-met and stat3 increased. By contrast, in hSulf-1-transfected HepG2 cells, the phosphorylation of c-met and stat3 decreased (Fig. 2E).

### hSulf-1 inhibits the proliferation of HepG2 cells through stat3 signaling

**Figure 3:**
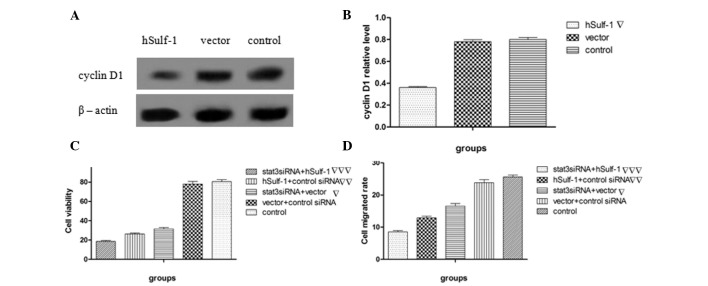
Effects of hSulf-1 on HepG2 cell proliferation and migration. (A and B) Cyclin D1 expression was examined by western blotting and then analyzed by densitometry. Bands were normalized with the corresponding β-actin density. (C) Cell viability was measured by MTT assay. The absorbance at 490 nm was measured using a microtiter plate reader and the viable cell number was calculated as a percentage. (D) Parental HepG2 cells, HepG2 cells transfected with the hSulf-1 expression vector, HepG2 cells transfected with the empty vector or control siRNA, HepG2 cells transfected with stat3 siRNA and the hSulf-1 expression vector, and HepG2 cells transfected with stat3 siRNA and the empty vector were seeded in transwell plates at a density of 5×10^5^ cells/ml. Cells that migrated through the Transwell were quantified by Transwell migration assay. ^▽^P<0.05, vs. control or vector groups; ^▽▽^P<0.05, vs. control, vector or stat3 siRNA groups and ^▽▽▽^P<0.05, vs. remaning groups. hSulf-1, human sulfatase-1; stat3, signal transducer and activator of transcription 3.

The effects of the stable transfection of hSulf-1 into hSulf-1-negative HepG2 cells on cell proliferation was examined and measured by MTT assay. The viability of hSulf-1-transfected cells was significantly decreased compared with that of vector-transfected cells (Fig. 3C). To confirm whether the stat3 signaling pathway is involved in mediating these inhibitory effects on cell proliferation, cells were also transfected with stat3 siRNA to knockdown the expression of stat3. The results showed that the inhibitory effects of hSulf-1 on cell proliferation were decreased following the knockdown of stat3 (Fig. 3C). Cyclin D1 protein, a protein involved in proliferation downstream of stat3, was also expressed at extremely low levels following transfection with hSulf-1 (Fig. 3A and B). In addition, following the knockdown of stat3 in hSulf-1-transfected cells, cell viability was further decreased (Fig. 3C). These results suggested that hSulf-1 inhibits HepG2 cell growth by suppressing stat3 signaling.

### hSulf-1 inhibits the migration of HepG2 cells

To further elucidate the relevance of hSulf-1 function in HCC, the effects of hSulf-1 on migration were investigated by Transwell chamber assay. The results showed that hSulf-1 transfection inhibits cell migration ability compared with vector transfection alone. Additionally, stat3 siRNA transfection decreased the migration of HepG2 cells and cells doubly transfected with stat3 siRNA, and the hSulf-1 expression vector exhibited further reductions in migration (Fig. 3D). These results suggested that hSulf-1 may affect stat3-mediated migration.

### hSulf-1 induces G_0_/G_1_ phase cell cycle arrest through stat3 signaling and promotes G_2_/M phase cell cycle arrest in HepG2 cells

**Figure 4:**
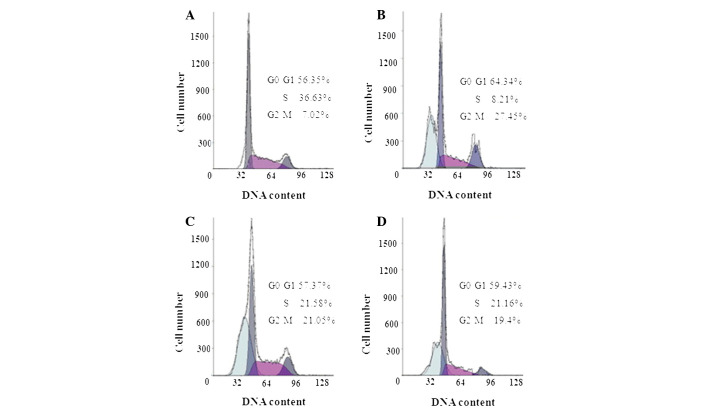
hSulf-1 induces G_0_/G_1_ and G_2_/M phase cell cycle arrest and promotes cisplatin-induced apoptosis in HepG2 cells. Cells were stained with propidium iodide and DNA content was analyzed by flow cytometry. The cell number in each phase in every group was calculated using ModFit software. HepG2 cells were transfected with (A) empty vector and control siRNA, (B) stat3 siRNA and empty vector, (C) hSulf-1 expression vector and control siRNA and (D) hSulf-1 expression vector and stat3 siRNA. hSulf-1, human sulfatase-1; stat3, signal transducer and activator of transcription 3.

Next, it was investigated whether the antiproliferative activity of hSulf-1 in HepG2 cells correlates with cell cycle arrest. As demonstrated in Fig. 4A–D, cell cycle analysis revealed that, compared with the vector and control siRNA groups, stat3 siRNA-transfected cells exhibited an increased number of cells in the G_0_/G_1_ phase, while the number of cells in the G_2_/M phase did not change. By contrast, when hSulf-1 was transfected into HepG2 cells, the number of cells in the G_0_/G_1_ and G_2_/M phases was increased. Therefore, it was assumed that hSulf-1 induces G_0_/G_1_ phase arrest in HepG2 cells through the stat3 signal pathway and also promotes G_2_/M phase arrest.

### hSulf-1 promotes cisplatin-induced apoptosis in HepG2 cells

**Figure 5:**
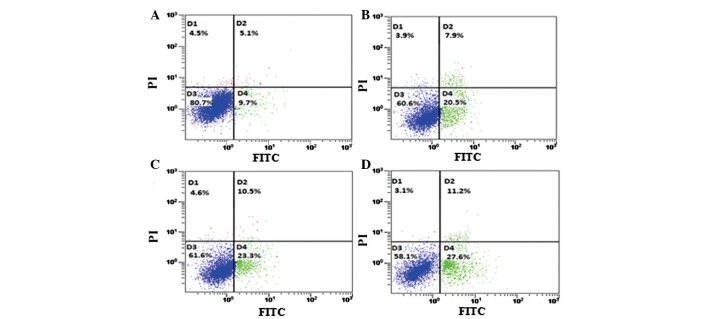
hSulf-1 promotes cisplatin-induced apoptosis in HepG2 cells. Apoptotic cells were assessed by qualitative flow cytometry using Annexin V/propidium iodide staining. The cells were as follows: D1, mechanically damaged; D2, late apoptotic or necrotic; D3, viable; and D4, early apoptotic. HepG2 cells were transfected with (A) empty vector and control siRNA, (B) stat3 siRNA and empty vector, (C) hSulf-1 expression vector and control siRNA and (D) hSulf-1 expression vector and stat3 siRNA. hSulf-1, human sulfatase-1; stat3, signal transducer and activator of transcription 3.

**Figure 6:**
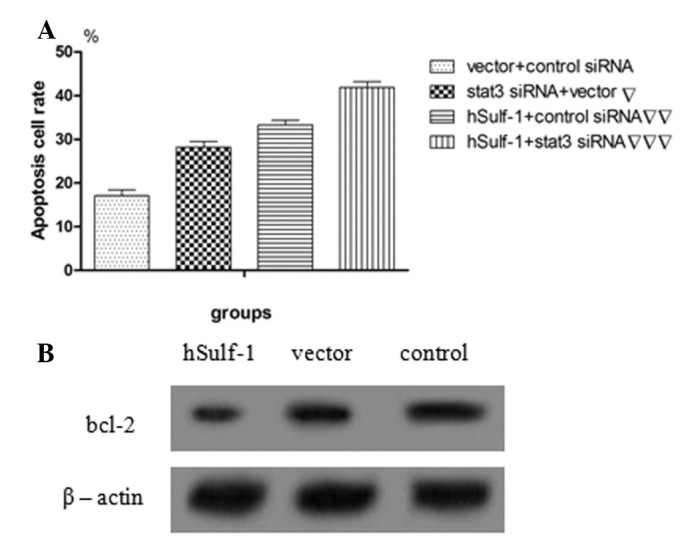
Apototic cell rate and expression of bcl-2 in HepG2 cells. (A) Apoptotic cells were calculated by qualitative flow cytometry. ^▽^P<0.05, vs. vector group; ^▽▽^P<0.05, vs. vector group or stat3 siRNA group; and ^▽▽▽^P<0.05, vs. remaining groups. (B) Expression of bcl-2 in vector-transfected HepG2 cells was increased compared with that in hSulf-1-transfected HepG2 cells. hSulf-1, human sulfatase-1; stat3, signal transducer and activator of transcription 3.

Dysregulation of cell growth and apoptosis is considered to lead to carcinogenesis. Therefore, the effects of hSulf-1 on cisplatin-induced apoptosis were examined in HCC cells using Annexin V/PI double-staining. hSulf-1-transfected HepG2 cells showed a higher sensitivity to cisplatin-induced apoptosis compared with that in control cells. Furthermore, the apoptosis rate in HepG2 cells was decreased following transfection with stat3 siRNA (Figs. 5A–D and 6A). Consistent with these observations, the expression of the antiapoptotic protein, bcl-2, which signals downstream of stat3, was found to increase in vector-transfected HepG2 cells compared with that in hSulf-1-transfected HepG2 cells (Fig. 6B). The results showed that the hSulf-1 expression promotes cisplatin-induced apoptosis in HepG2 cells and correlates with stat3 signaling to a certain degree.

## Discussion

Previous studies have reported that the hSulf-1 protein is an arylsulfatase that negatively regulates the sulfation of HSPGs (5,26–27). Notably, hSulf-1 desulfates cell surface HSPGs and subsequently downregulates receptor tyrosine kinase signaling. Therefore, hSulf-1 may be considered a tumor suppressor gene (8,12,28). hSulf-1 also affects the binding of heparin-binding factors to their receptors in several signaling pathways and suppresses the phosphorylation and activation of receptor tyrosine kinases. However, its molecular mechanisms are not well known. The stat3 signaling pathway, which may be activated by several growth factor receptors, such as EGFR and PDGFR (19–20), is known to be associated with the progression of HCC; thus, the effects of hSulf-1 on stat3 signaling must also be explored in HCC cells. The current study demonstrated that hSulf-1 expression is downregulated in HCC cell lines, including HepG2, Hep3B, Huh-7 and SMMC-7221. In various types of cancer, DNA methylation and histone modification are involved in gene regulation. The present study demonstrated that DNA methylation and histone modification regulate hSulf-1 expression that synergistically effects the demethylating agent and histone deacytelase inhibitor, resulting in the expression of hSulf-1. This indicated that epigenetic modifications of DNA and histones are a mechanism of hSulf-1 inactivation and other mechanisms involved in the interaction between DNA methylation and histone modification in HCC. In addition, the link between hSulf-1 and stat3 signaling was further investigated. The results revealed that hSulf-1 inhibits cell proliferation and migration, induces G_2_/M phase cell cycle arrest and promotes apoptosis through the suppression of stat3 signaling in HepG2 cells. To verify the negative effects of hSulf-1 on cancer angiogenesis, hSulf-1-expression vector and stat3 siRNA were constructed. hSulf-1 expression was found to downregulate the phosphorylation of stat3, but had no effect on total stat3 expression, indicating that hSulf-1 regulates the activity of stat3.

HGF is a key regulating factor in cell proliferation, motility and differentiation. We first hypothesized that the stat3 signaling pathway may be activated by HGF, similar to other growth factors. Subsequently, it was determined whether hSulf-1 may mediate the HGF-dependent stat3 signal pathway. Following HGF treatment, the phosphorylation of stat3 and the expression of c-met decreased in hSulf-1-transfected HepG2 cells, indicating that hSulf-1 suppresses the phosphorylation of stat3, which is mediated by HGF. This effect may correlate with the activity of receptor molecules since c-met activates the phosphorylation of stat3. However, when hSulf-1 was re-expressed following transfection with the hSulf-1 expression vector, c-met expression was also inhibited, thereby further influencing the phosphorylation of stat3.

In addition, the current study investigated the correlation between hSulf-1 and stat3 signaling on the effects of cell proliferation, migration and apoptosis. hSulf-1 has been previously shown to inhibit cell growth and invasion through HGF, FGF, HB-EGF, VEGF and wnt signaling (6,12,29–30). In the present study, when HepG3 cells were transfected with stat3 siRNA to silence the expression of stat3, cell viability and motility were decreased to a certain extent. This effect was more apparent following the additional transfection of the HepG2 cells with hSulf-1, suggesting that hSulf-1 is involved in the regulation of cancer cell proliferation and migration, partly due to the inhibition of stat3 signaling. Consistent with these observations, the expression of cyclin D1, a downstream effector of stat3 signaling in the regulation of cell proliferation (31), decreased in hSulf-1-transfected HepG2 cells. These results demonstrated that stat3 activation in cancer cell proliferation and migration is mediated by the effects of hSulf-1.

Next, the present study investigated whether the antiproliferative activity of hSulf-1 in HepG2 cells was due to cell cycle arrest and apoptosis. hSulf-1 was found to induce G_0_/G_1_ arrest and apoptosis partly through the stat3 signaling, and to also promote G_2_/M phase arrest. The results of the Annexin V/PI double staining demonstrated that hSulf-1 transfection increases the number of apoptotic HepG2 cells. Previous studies have revealed that stat3 signaling is pivotal in the antiapoptotic process, mediated through downstream proteins, including bcl-2 and bcl-XL, which allow cells to resist apoptosis (32–33). Additionally, stat3 signaling has been shown to promote G_0_/G_1_ phase cell cycle arrest (34–35). In the current study, cisplatin treatment also induced G_0_/G_1_ phase arrest and downregulation of stat3 signaling in HepG2 cells. Therefore, the effects of hSulf-1 on apoptosis and G_0_/G_1_ phase cell cycle arrest may involve downregulation of the stat3 pathway. Furthermore, it was found that hSulf-1 expression alone induces G_2_/M phase arrest in HepG2 cells; elucidation of the mechanisms involved requires further investigation.

In conclusion, the results of the present study demonstrated that hSulf-1 re-expression attenuates the phosphorylation of stat3, suppresses cell proliferation and motility and promotes cancer cell apoptosis. These effects correlate with the downregulation of stat3 signaling in HepG2 cells. Thus, the study provides a novel insight into the molecular mechanisms of hSulf-1 in HCC cells. These observations strengthen the theory that hSulf-1 is a promising target for therapeutic intervention through the stat3 signaling pathway in HCC.
